# No Country for Asylum Seekers? How Short-Term Exposure to Refugees Influences Attitudes and Voting Behavior in Hungary

**DOI:** 10.1007/s11109-021-09682-1

**Published:** 2021-02-09

**Authors:** Theresa Gessler, Gergő Tóth, Johannes Wachs

**Affiliations:** 1 Department of Political Science, University of Zurich, Zurich, Switzerland; 2 Spatial Dynamics Lab, University College Dublin, Dublin, Ireland; 3 Agglomeration and Social Networks Research Lab, Centre for Economic and Regional Studies, Budapest, Hungary; 4 Institute for Data, Process and Knowledge Management, Vienna University of Economics and Business, Vienna, Austria; 5 Complexity Science Hub Vienna, Vienna, Austria

## Abstract

How does exposure to refugees influence political behavior? We present evidence from Hungary, a country with widespread anti-immigration attitudes, that short term exposure during the 2015 refugee crisis predicts anti-refugee voting and sentiment. We code exposure to refugees at the settlement level using reports from state media, an independent online news site, and an online social media aggregator. Settlements through which refugees traveled showed significantly higher anti-refugee voting in a national referendum in 2016. The effect decreases sharply with distance. Using a difference-in-differences model, we find that the far-right opposition gained, while the governing right-wing party lost votes in these settlements in subsequent parliamentary elections. This suggests incumbents are punished by voters skeptical of immigration regardless of their policy position. Survey data supports this finding of a competition among right-wing parties, as individuals in exposed settlements are more fearful of immigrants and support restrictive policies only if they identify as right-wing.

The issue of migration has moved to the core of the European political conversation since the 2015 refugee crisis. It is likely to stay on the agenda as subsequent years have set new records for the highest number of people forcibly displaced and searching refuge on record (UNHCR 2020). Despite the broad political salience of the issue, the local circumstances framing the encounters that natives have with refugees and immigrants vary widely. For instance refugees may seek to settle in a country or may just be passing through. In the latter case we can expect greater geographic differences in the likelihood of natives encountering refugees and that interactions between natives and refugees are brief and one-time. In such situations, political elites including governments have a critical role: some scapegoat immigrants rather than sanctioning positive engagement. Thereby, they can frame the context in which refugees make first impressions on voters in a negative light (Harteveld et al. 2017; Ivarsflaten 2005). As populist and radical right parties hostile to immigration become more successful and increasingly participate in government (Kaltwasser et al. 2017; Bustikova 2017), it is important to observe how voters react to nativist appeals when they come from parties in government, and how such responses are conditional on previous political attitudes and actual encounters with refugees.

In this paper we present evidence that exposure to refugees during the 2015 European refugee crisis affected political behavior in Hungary, a country at the center of the crisis, given the large number of refugees entering the country and its government’s nativist response. The Hungarian case differs from those used in recent work to examine the effect of contact with refugees on native political behavior (Hangartner et al. 2018; Dinas et al. 2019; Steinmayr 2020) because refugees were passing through the country in an irregular manner and their interactions with locals were highly transient. Indeed, because of staggered border closures, changing opportunities to travel by train or bus, and spontaneous decisions of groups of refugees to try to reach Austria on foot, many Hungarian settlements were only exposed to refugees on a single occasion. Another unique aspect of the Hungarian case is that the ruling Fidesz party, led by Viktor Orbán, mobilized radically against the refugees in the aftermath of the crisis. Migration became the central question of Hungarian politics in subsequent years (Krekó and Enyedi 2018). The share of Hungarians who named immigration as one of the country’s most important problems increased from close to zero in mid 2013 to over thirty percent in November 2015 (European Commission 2016) and has remained high ever since.

Shortly after the crisis, Hungary held a national referendum on proposed EU refugee quotas. We use this vote to measure the effect of short-term contact with refugees on voting behavior at the settlement (municipality) level. The results of the referendum allow us to more directly measure anti-refugee sentiment than previous studies which use far-right party outcomes as a proxy (Dinas et al. 2019; Steinmayr 2020; Dustmann et al. 2018; Vertier and Viskanic 2018). We find a significant backlash effect: settlements exposed to the crisis were significantly more likely to vote against the EU quota in the referendum.

Though Fidesz won a significant victory in the 2018 parliamentary elections with a campaign centered on the topic of migration, we observe an interesting difference in voting trends from the 2014 elections among exposed and non-exposed settlements. In settlements exposed to the refugee crisis, there was a small but significant move from Fidesz towards the far-right opposition Jobbik party, which also took a hardline anti-refugee stance during the campaign. This suggests that beyond the issue-ownership theory favored by previous work (Dinas et al. 2019), there is an anti-incumbent aspect to anti-refugee voting induced by exposure to crisis which ruling parties cannot exploit. Voters inflamed by direct exposure seem to punish the ruling party  (see also Bratti et al. 2020).

We find further evidence that exposure influences voting behavior only among right-wing voters using survey data collected between the crisis and referendum. We confirm that individuals from exposed settlements are more likely to express anti-refugee policy preferences, but find that this is conditional on voting for parties on the right.

We proceed by outlining a theoretical framework for understanding the effect of exposure to the refugee crisis on voting behavior and reviewing related work. After describing the specifics of our case, we present our data and modelling strategy. We then proceed to test the impact of short-term exposure to refugees in different settings: its effect on voting in the national anti-refugee referendum, the electoral outcomes of two anti-refugee parties (one in government, one in opposition) in parliamentary elections before and after the crisis, and individual-level survey responses collected shortly after the crisis.

## Motivation, Theory and Related Work

The so-called 2015 European refugee crisis has led to renewed interest in how natives react to the arrival of immigrants and refugees both in terms of attitudes and political behavior. The crisis has drastically increased the number of arrivals to Europe and changed patterns of interactions between natives and new arrivals. While many classic studies build on the *contact theory* by Allport (1954) which posits that social interactions can lead to a reduction of prejudices  (see also: Pettigrew et al. 2011; Paluck et al. 2018), the conditions for sustained contact were certainly not met in Hungary and many other countries during the crisis. As in other so-called transit countries, refugees moved on as soon as they were able to, often only spending days or even hours in a country. We suggest this time was too short to overcome barriers of language and culture.

While data from Eastern European countries on the route taken by the refugees is limited, evidence from other regions supports this notion: in a study of reactions to refugees in Austria, Steinmayr (2020, p. 23) argues that the arrival of refugees to settlements created substantial anxiety which reduced only after refugees had lived in the respective settlement for some time. Although prejudice may be moderated in the long run, short and involuntary encounters may even inflame prejudice (on the difference between short- and long-term effects: Enos 2014). Hence, Hungary presents an ideal case to study the conditions under which short-term encounters may have long-lasting consequences.

In explaining why and when citizens may perceive refugees as a threat in the US context, Hopkins (2010) shows that reactions to immigrants are most likely to be hostile when communities experience a sudden influx of immigration and when national media rhetoric presents this as a threat. He argues that citizens are typically unaware of immigration levels but that they are particularly sensitive to changes to these levels, which he finds may lead to politicization of the topic (Hopkins 2010, p. 42). In this case, local arrivals and hostile national rhetoric combine to produce negative reactions to refugees.

In this context Hungary provides an interesting case: a significant amount of refugees passed through the country in summer and autumn 2015 on their way to Western Europe, until the borders were sealed by a physical barrier in the fall. The topic dramatically gained salience for voters as the share of Hungarians who named immigration as one of the country’s most important problems increased from close to zero in mid 2013 to over thirty percent in November 2015 (European Commission 2016). While Hungary fits the situation outlined by Hopkins (2010) regarding the salience of anti-immigration rhetoric (Bocskor 2018), exposure in most places was temporary. In many cases, refugees merely passed settlements on their way through the country. Indeed the overall level of foreigners living in Hungary has not changed significantly in the period from 2008 (175,000) to 2019 (180,000) (Hungarian Central Statistical Office, 2019). This situation provides a test of the effects observed by Hopkins with a key difference: a subsequent return to the previous level of immigrants.

We suggest that this reversion to the status quo does not change the substantive effect on political behavior of residents of Hungarian settlements exposed to the crisis. One likely contributing factor is the strong anti-refugee message in the public discourse in the years following the crisis: the manner in which governments address the issue of immigration has consequences for citizens’ attitudes on the issue (Hainmueller and Hopkins 2014). Voters are susceptible to elite opinion leaders who are skeptical towards immigration more generally (Ivarsflaten 2005). In this, Hungary is an extreme case as Hungary’s governing elites actively promoted fears of refugees, for example by evoking the idea of an “invasion”. The governing party and the most popular opposition party at the time espoused anti-refugee positions, while Hungarian media rarely gave refugees a voice (Bernáth and Messing 2016). As individuals interpret their personal experiences through the lens of public discourse, short-term encounters, especially with groups of refugees, will reify the framing of refugees as dangerous. Moreover, such encounters are likely to increase the salience of immigration for citizens which in turn may have important behavioral consequences (Dennison 2019).

Several other studies have analyzed the political outcomes of the recent refugee crisis, albeit with different results. Evidence from France (Vertier and Viskanic 2018) and Austria (Steinmayr 2020) exploiting quasi-random refugee settlement programs find support for the contact hypothesis in the context of long-term contact. In both countries, settlements receiving refugees were less likely to vote for the far-right in subsequent elections. Short-term exposure during the crisis has been studied using data from the Greek islands. Dinas et al. (2019) find an increase in the vote share of the far-right Golden Dawn on islands exposed to the refugee crisis. Hangartner et al. (2018) find more negative attitudes towards refugees on the same islands in a survey fielded almost two years later.

The Hungarian case presents an opportunity to revisit two lines of research about the effect of short term exposure on political behavior and to address gaps therein. One issue with previous works cited above is that they measure change in voting behavior using presidential or parliamentary votes for right-wing parties (Dinas et al. 2019; Steinmayr 2020; Dustmann et al. 2018; Vertier and Viskanic 2018). Though anti-immigration is a uniting element of right-wing party ideologies in Europe (Ivarsflaten 2008), citizens may vote for them for other reasons, for example because of their culturally conservative programs. As Hungary held a national referendum on a refugee related policy question shortly after the crisis, we can examine the relationship between exposure during the crisis and anti-refugee voting attitudes more directly through voting behavior. We assume experiences with refugees in local contexts serve as reference, tying the crisis to everyday life. Even if their presence was transient and contact limited, images and anecdotes of unfamiliar refugees in familiar places will later influence political attitudes on immigration. This familiarity does not stop at the borders of individuals’ own settlement but also includes their immediate surroundings and places residents frequently travel to. The media’s intense coverage of the refugee crisis meant Hungarians also saw images of refugees in neighboring settlements, even if they and their immediate social contacts did not directly witness the incident. All Hungarians were exposed to the outlined negative rhetorical imagery. Whether citizens living in settlements near the refugee routes personally saw refugees, heard about them through their social networks, or saw what happened on state television, their familiarity with the setting personalizes the events. Hence, we expect the effect of exposure to go beyond the location of exposure itself and include nearby settlements.

1. Hungarian settlements exposed to refugees during the crisis are more likely to vote against refugee resettlement quotas in the 2016 referendum. The effect extends to nearby settlements but decreases sharply with distance.

A second point of interest in this line of research we reevaluate is whether exposed voters alter their voting behavior to punish the government or to support anti-immigration policy. So far, studies can only indirectly control for this, e.g. by looking into the electoral gains by other opposition parties. For example, Dinas et al. (2019) argue that votes for the Golden Dawn, a far-right opposition party in Greece, are policy-votes rather than anti-government votes. In Hungary we can disentangle this question by comparing the change in vote shares of Fidesz, the governing right-wing party, and Jobbik, a far-right opposition party, across parliamentary elections from 2014 and 2018.

We suggest that the policy aspect is not the main driver of the vote for the radical right in settlements exposed to the crisis. A key aspect of right parties’ capacity to benefit from short-term exposure may be due to disappointment with governing parties. A study from Italy indicates that settlements hosting more refugees were significantly less likely to support Matteo Renzi’s proposed constitutional amendment, a referendum that had no direct link to the refugee crisis (Bratti et al. 2020). While right-wing parties may benefit disproportionately, studies of right-wing populist parties have shown these parties frequently claim that governing elites prioritize the interest of immigrants above those of the native population (Mudde and Kaltwasser 2017, 14; de Cleen 2017, 350). More generally, it is difficult for governing parties to shift responsibility for immigration under their watch. While Fidesz attempted to solve this dilemma by adopting a tough stance on immigrants and trying to physically constrain immigrants to few places, exposed settlements were the few places that nevertheless experienced the refugee crisis directly. Thus, citizens in these settlements may be discontent with the government’s handling of the immigration crisis and cast their ballot for the opposition Jobbik instead. Hence, we argue that policy considerations shape a voting behavior that is ultimately motivated by disappointment with incumbents.

H2: Jobbik, the anti-refugee party in opposition, gained votes relative to Fidesz, the anti-refugee party in government, in settlements exposed to the crisis.

Given this hypothesis of a reshuffling of voters on the right, it is natural to ask if there is heterogeneity in the effect of exposure based on partisanship. If exposure also changes the attitudes of left-wing voters towards immigration, Fidesz may ultimately benefit from a strengthening of the right camp, even if the party loses some voters to its opposition competitor Jobbik.

We suggest that the effect of exposure is conditioned by political attitudes. We do so, in part because of the strongly partisan nature of the immigration issue: Evidence suggest that voters adjust their views on immigration to the position of their party (Harteveld et al. 2017) and individuals may resort to motivated reasoning based on partisan ideology in their interpretation of experiences with immigrants. Similarly, issue salience, an important predictor of vote choice and potentially relevant for attitudes, also varies for citizens dependent on their party attachment (Neundorf and Adams 2018; Dennison 2019).

While it is unclear if the effect of exposure conforms to such partisan pressures, prior evidence provides us with reasons to think so: recent evidence suggests that inaccurate perceptions about the size of foreign-born populations are a consequence of anti-refugee attitudes, and not their cause (Hopkins et al. 2018). Thus, citizens who are sceptical towards immigration may experience the refugee crisis as more threatening.^1^ To borrow a term from Sniderman et al. (2004), we posit that short exposure *galvanizes* constituencies already concerned with the topic. Given that policy on immigration and refugees in Hungary is a significantly partisan issue and has become increasingly so during the crisis, we propose that the anti-refugee reaction of citizens to exposure to refugees during the crisis is a right-wing phenomenon.

H3: The effect of refugee exposure on political behavior depends on an individual’s political attitudes. Short-term exposure is more likely to induce anti-immigrant sentiments in right-wing voters.

## The Hungarian Case

Hungary is more ethnically homogeneous than most other European countries. The most common immigrants to Hungary are ethnic Hungarians coming from neighboring countries. Since 1990 immigration to Hungary has functioned, both formally and informally, as a two track system distinguishing between ethnic Hungarians and other immigrants (Nyíri 2003; Bocskor 2018). This framework reflects the negative Hungarian attitude towards refugees in particular and non-Hungarian immigrants in general (Simonovits et al. 2016; Enyedi et al. 2005; Messing and Ságvári 2016). Immigration of non-Hungarians was not previously a significant topic in Hungarian politics. However, nation and nationality were salient topics in other regards e.g. the question of citizenship for ethnic Hungarians from abroad (Batory 2010). While certain ethnic groups certainly have advantages in questions of immigration in all European states, the institutionalized two-tier system in Hungary facilitates xenophobia, for example against the small Chinese and Vietnamese immigrant communities (Nyíri 2003). Indeed, in a comparative analysis using the European Social Survey, Bail (2008) finds that in Hungary symbolic boundaries, conceptual distinctions used by majority groups to construct notions of “us” and “them”, have the strongest racial component of all 21 countries. This fertile ground of ethnic prejudice may have been amplified by media reporting about the crisis, similar to how anti-Roma discourse has entered the mainstream (Vidra and Fox 2014).

### 2015 Refugee Crisis

The importance of immigration as a political issue in Hungary changed drastically in 2015, as rising immigration numbers and attacks in western Europe led to the political mobilization of the topic on the right. Hungarian Prime Minister Viktor Orbán began to frame immigration as a threat to Hungary in January 2015 in the aftermath of the attack on Charlie Hebdo. The government mailed a “national consultation” questionnaire to each Hungarian citizen on the subjects of immigration and terrorism. The questionnaire was criticized for its leading questions and its framing of the issue^2^.

**Fig. 1:**
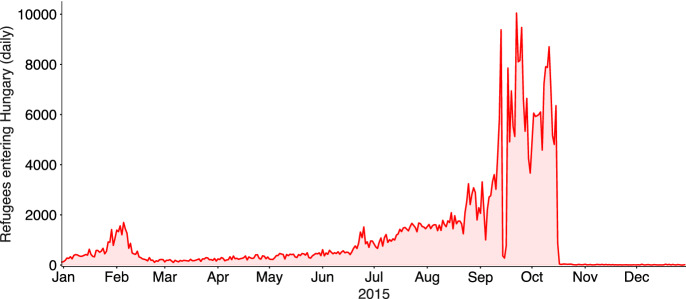
Number of refugees entering Hungary daily in 2015. The Serbian border was sealed on September 18th, causing a brief, sharp decrease in entries. The Croatian border was sealed on October 17th, practically ending the inflow of refugees to Hungary. *Source: police.hu - Border information*

While immigration numbers had been on the rise since 2014, it was only in summer 2015 that refugee traffic reached its high point and that the issue gained traction with the wider public. As Hungary was the first Schengen country besides Greece before destination countries like Austria and Germany on the so-called Balkan Route, a land route taken by refugees from Greece, nearly 400,000 refugees were registered in Hungary in 2015. Most arrived in August, September and October and were not able to continue their journey at first, due to the EU’s Dublin Regulation which required refugees to apply for asylum in the first Member State they reached. We visualize the number of refugees entering Hungary in 2015 in Fig. 1. The majority of these refugees entered at the Serbian border, making this area a frequent focus of public debate.

The patterns of movement of refugees through Hungary during the crisis was highly irregular. While most refugees entered the country at border crossings with Serbia and then later Croatia, they took many different routes to reach Austria depending on the stage of the refugee crisis. Some refugees were taken to camps in different parts of the country by bus or train. Others managed to get to Budapest, the transportation hub on the way to Austria, but were then blocked from proceeding. In one memorable case, a group of refugees in Budapest boarded a train to a refugee camp in Bicske, thinking that it was going to Vienna. They refused to disembark and remained in Bicske for some time (Kallius et al. 2016). We highlight additional examples of irregular movement when we introduce our measure of refugee contact. For now we emphasize that the dynamically changing regulations consistently scrambled the routes taken by the refugees.

This observation suggests two ways in which the Hungarian case is distinguished from the experience on Greece’s so-called “hot-spot” islands. First, geography more significantly determined which islands became hot-spots in Greece, while volatility in policy was a more significant factor in explaining where refugees went in Hungary. Second, while both Greek and Hungarian natives typically had transient or one-off encounters with individual refugees, the Greek hot-spots were more persistent over time, with new refugees passing through the same places for an extended period of time. In the Hungarian case, refugees were only present in most specific locations for a short amount of time.

Despite the transient nature of the crisis, the issue of migration remained important. This is because for the Hungarian government, decreasing migration became a central goal. This was realized through the construction of a fence along the borders with Serbia and Croatia. When the fence along the Serbian border was completed on September 18th 2015, the Hungarian authorities closed the border, diverting the refugees through Croatia. One month later, that border was closed too. Afterwards, very few refugees entered Hungary as the government drastically restricted the number of legal entries via so-called “transit zones” at the border. However, public discussion regarding how to deal with refugees and how to manage Hungary’s border has continued since then as the centerpiece of the ruling party’s political discourse.

### Political Consequences and the 2016 Quota Referendum

With its restrictive immigration policy and intensive mobilization around the issue (Bocskor 2018; Goździak and Márton 2018), the governing Fidesz party created a strong link between the prevailing political cleavages and immigration (Palonen 2018; Barna and Koltai 2019). Immigration had previously been a marginal issue in Hungarian party competition with cultural competition centered around nationalism and cultural liberalism (Gessler and Kyriazi 2019) in line with other Eastern European countries (Hutter and Kriesi 2019). After the 2014 election, Fidesz faced increasing pressure from the right, with the oppositional far-right party Jobbik gaining popularity (Batory 2016; Bustikova 2017). As Fidesz actively competed for a far-right electorate by enhancing policies that originated from Jobbik (Pirro 2018; Szalai and Göbl 2015), the immigration issue (on which there was no clear issue ownership given its low salience) provided fertile ground for an outbidding regarding restrictive policy proposals between both parties.

Originally, discussion centered around border security. After the closing of the borders, political discussion continued regarding the European level and the European Union’s proposed quota-based refugee allocation scheme. According to this scheme, Hungary would be responsible for hosting 1294 refugees. A referendum on the policy was originally proposed by Jobbik in parliament in November 2015, however, the proposal was not advanced. Fidesz also opposed the quota but only announced a referendum in February 2016, to be held in October, a year after refugee arrivals to Hungary had effectively ended. The question posed by the referendum was: “Do you want the European Union to be able to mandate the obligatory resettlement of non-Hungarian citizens into Hungary even without the approval of the National Assembly?” Despite the EU-centric phrasing of the question, the subsequent government campaign was centered on presenting immigration as a risk to the Hungarian population, criticizing the EU’s position rather than being anti-European (van Eeden 2019). Indeed, the government’s position was that it was defending Europe and European values and its campaign focused on the risks refugees present to public safety, cultural heritage, and the labor market in Hungary.^3^ Hungarian public perception of the EU has remained stable across the crisis and referendum (European Commission 2016).

Since the referendum required 50% participation to be valid, some opposition parties encouraged voters to stay home, others to cast invalid ballots. Ultimately, 41% of eligible voters cast a valid ballot and of those 98% voted “No”, i.e. against the EU quota. In our subsequent analysis of referendum outcomes across settlements, we will consider the ratio of no votes to the eligible voting population, that is, the ratio of citizens who followed the governmental recommendation.

Since then, the Hungarian government has held additional “national consultations” and the topic has remained on the agenda up to and beyond the 2018 parliamentary election (Krekó and Enyedi 2018; Bocskor 2018; Gessler 2017). Competition between Fidesz and Jobbik has remained a driving force of this conflict with both espousing policies to curb immigration. In the context of our study, this means both gained different credentials on the immigration issue: while Fidesz was able to build a track-record of implementing restrictive policies, Jobbik may at times have increased its profile by attacking domains in which Fidesz did not advance new policies, e.g. the country’s residency bond scheme that offers residence permits to foreign citizens in exchange for buying government bonds (Jacoby and Korkut 2016; Halmai 2017).^4^

## Data and Measurement

To test our hypotheses, we collected data on the presence of refugees in Hungarian settlements, specifically municipalities, during the peak crisis months in 2015 from three media sources. We relate this to political outcomes while controlling for several potential confounding factors at the settlement level. When using survey data to test heterogeneity of the treatment effect on individuals, we also employ individual-level controls.

### Exposure to Refugees

We collected data on the presence and movement of refugees during the crisis from three sources: MTI, the Hungarian state newswire, Index, a popular online news outlet independent from the government, and LiveUAMap (Live Universal Awareness Map), an NGO-run crowdsourced real-time social media aggregator with geographic information including geocoded pictures and videos. We choose MTI and index.hu since they are both reliable sources but are influenced by different political ideologies. Furthermore, both have a large reach: The reports of MTI as national newswire are used by many other news outlets, including local news stations. index.hu directly targets consumers and is one of the most visited pages in Hungary, averaging over 1.5 million daily views in 2018 (ITE.hu 2018). Though most of the activity on LiveUAMap relates to the conflicts in Ukraine and Syria, there is also significant amounts of data on the events of the European refugee crisis. It has been used in qualitative studies of the paths taken by refugees on their way to Europe (Proglio 2018).

**Fig. 2:**
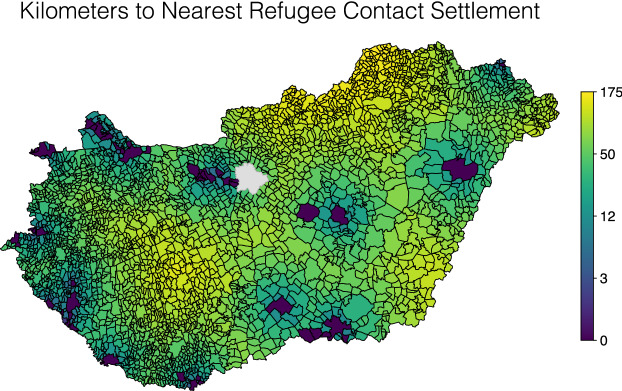
Settlement distances to points of exposure with refugees during the 2015 crisis, logarithmic scale. Budapest (in gray) is omitted

We coded that significant refugee exposure took place in a settlement if it was reported in any of the three sources. For example, we include all the settlements along the “March of Hope”, a widely reported incident often cited as the climax of the crisis in Hungary (Kallius et al. 2016). On September 4th, thousands of refugees at Budapest’s Keleti train station, which was closed to international travel because of the crisis, began walking towards Austria along the M1 highway, disrupting traffic on one of the largest highways in the country. Later that same evening, the Hungarian government decided to bus the refugees to the Austrian border. Soon afterwards, chancellor Angela Merkel signaled that the refugees would be allowed to come to Germany. We also coded smaller scale events throughout the country, including similar spontaneous marches from the Vámosszabadi refugee camp in the northwest to the Austrian border, and from the Croatian border to the train station in Nagykanizsa in the southwest. In total we label 51 settlements as treated. We visualize the geographic distribution of refugee exposure in Fig. 2.

Though we do not claim that we have identified every location of exposure of Hungarians to refugees during the crisis and acknowledge that neutral interactions between refugees and natives are more likely to be missing from our data, we do suggest that the data accurately describes those locations in which Hungarians had a significantly higher likelihood of seeing unfamiliar refugees in a familiar context. The intense public interest in the day-to-day events of the crisis, evidenced by Index’s use of a livestream format to disseminate updates, and our use of three heterogeneous sources minimize the risk of missing significant encounters. Indeed, survey data from January 2016 indicates that individuals in such settlements are significantly more likely to report having encountered a refugee in the past year. This relationship holds even when controlling for whether the individual reports knowing a foreigner personally and whether he or she lives near a border, see Table 7 in the Appendix. Finally, we note that we have excluded Budapest as datapoint from our empirical analysis because it is an outlier in several dimensions including population, density, diversity, and wealth, and because treatment in the city itself was highly heterogeneous.

### Dependent Variables

In our empirical analysis we analyze three different types of political outcomes: settlement-level outcomes of the quota referendum, settlement-level election results in the following general election in April 2018, and individual responses to a survey on migration-related topics conducted in January 2016. We report summary statistics of all variables used in our models in the Appendix, see Tables 4 and 6.

**Fig. 3:**
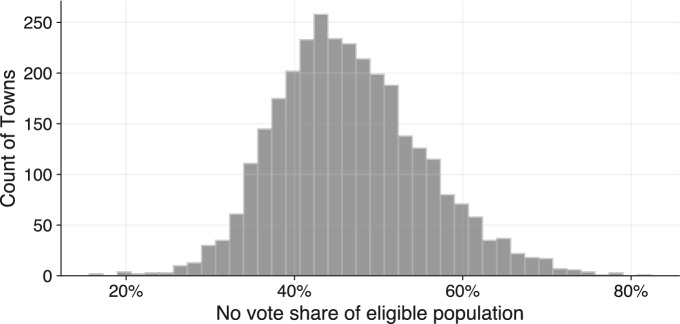
Distribution of refugee quota referendum no votes as share of the eligible voting population of Hungarian settlements with at least 50 voters

We plot the distribution of our primary dependent variable, the ratio of no votes cast in the referendum to the eligible voting population in a settlement, in Fig. 3. Given the boycott strategy of the opposition discussed before, this is a more appropriate measure of the anti-refugee outcome than considering the share of votes against the quota. We note that there is significant variance between settlements (IQR: 41–52%, excluding Budapest). We visualize the geographic distribution of the referendum outcomes in Fig. 5 in the Appendix. In a second specification, we measure the electoral effects of exposure to refugees on party outcomes at the settlement level. Immigration was a major topic of the 2018 election particularly for Fidesz and Jobbik, leading us to use the gains of Fidesz, Jobbik, and both combined as dependent variables.

In the individual-level specification, we use data from a representative survey of the general population of Hungary in January 2016. Specifically, we rely on a rotating module of a repeatedly asked questionnaire of TARKI, a Hungarian social research institute. After excluding respondents from Budapest, we are left with a sample of 772 respondents, 105 of which live in treated settlements. As for the analysis of electoral outcomes, we excluded Budapest because of its outlier status and the greater potential for heterogeneity of treatment within the city. We report similar though slightly weaker effect sizes when including Budapest in the Appendix.

We analyze a battery of attitudinal and policy questions that are included in the Appendix and discussed in more detail in the results section. Given the skew of the answers towards anti-refugee attitudes, we dichotomize responses into absolute rejection and more moderate attitudes.

### Control Variables

We collected socio-economic data for all Hungarian settlements to rule out some potential confounding factors. Many studies have shown that economically vulnerable populations are more likely to vote for radical right and anti-immigrant parties (Betz 1994; Fitzgerald and Lawrence 2011). Lower levels of education have also been shown to relate to political hostility towards foreigners (Hjerm 2001). We therefore control for each settlement’s income per capita, unemployment rate, and share of population with a high school degree in 2016, the year of the referendum. We also include logged population size to control for the size of the settlement.

Additionally, we consider voting data from the previous parliamentary elections in 2014 to account for prevailing local political allegiances. Because they both endorsed and campaigned for the anti-quota ’no’ camp, we include the share of votes received by Jobbik and Fidesz in 2014 in our models.

As we are also interested in potential spillovers of the exposure effect to nearby settlements, we use a matrix of inter-settlement travel distances (in minutes by car) to calculate the distance of each settlement to the nearest point of refugee exposure^5^. As issues of migration may be more salient near borders, we also note if a settlement is within 25 kilometers of a border.

### Estimation Strategies

To measure the anti-refugee sentiment at the settlement level we use the ratio of ’no’ votes to the eligible voting population in the 2016 referendum as a dependent variable $Y_i$, the distribution of which we show in Fig. 3. $T_{i}$ is a dummy variable with a value of 1 if we code refugee exposure in a settlement, $Z_{i}$ denotes our matrix of settlement-level control variables, including pre-referendum settlement-level party preferences, population, and socio-economic factors. $\epsilon _{i}$ is an independent error term, assumed normally distributed with mean 0. In the first extension of the baseline model, we introduce a geographical dummy $D_{i}$ for settlements within 25 kilometers of any border and county fixed effects $\psi _{i}$ to control for geographic effects like different settlement structures.

1 $$\begin{aligned} Y_i=\alpha _{i}+\delta _{1}T_{i}+\beta _{2}Z_{i}+\beta _{3}D_{i}+\psi _{i}+\epsilon _{i} \end{aligned}$$

We also measure the spillover effect of the treatment to nearby settlements using continuous distance measures to the nearest treated settlement in travel minutes. In order to examine the effect of treatment in terms of distance, our final model estimating a settlement’s referendum outcome bins observations into categories according to their distance in travel time from the nearest treated settlement, with treated settlements taken as the reference category.

To address the electoral effects of the refugee crises on parties we use a difference-in-differences estimation strategy. Specifically, we measure the effect of treatment during the crisis on vote shares of right-wing parties between the 2014 and 2018 Hungarian parliamentary elections. The specification constructs a counterfactual estimation of the change in vote shares in treated settlements using changes in vote shares in untreated settlements over the same period. The main threat to a causal interpretation of the resulting estimates would be a violation of the parallel trends assumption that party vote shares would have followed the same trend in all settlements had the refugee crisis not occurred. To address this concern, namely to assess whether the parallel trends assumption holds, we carry out and report a placebo test for differences in party vote shares between the 2010 and 2014 elections.

We also use a kernel-based propensity matching strategy (d’Agostino 1998; Stuart et al. 2014) to compare settlements using the same demographic and socio-economic controls as in the ordinary least squares (OLS) specifications. Specifically, we estimate the following model:

2 $$\begin{aligned} Y_{it}=\alpha _{i}+\alpha _{t}+\beta _{1}A_{it}+\beta _{2}(T_{it}|\omega _{i}) +\delta _{1}[A_{it}*(T_{it}|\omega _{i})]+Z_{i}+u_{it}, \end{aligned}$$

where the dependent variable is the vote share of the two main right-wing parties, $A_{it}$ is an indicator separating time periods before the refugee crisis ($A=0$) from the period after the crisis ($A=1$), and $T_{it}$ is the separation of settlements according to exposure to refugees as defined above.

The key variable of interest is the interaction term between $A_{it}$ and $T_{it}$, which estimates the true treatment effect. $Z_{i}$ refers to the socio-economic control variables, while $\omega _{i}$ is the matching estimator. When using kernel matching, each treated observation *i* is matched with several control observations, with weights inversely proportional to the distance in propensity scores between treated and control observations. The propensity scores are estimated using a logit regression using the same controls.

Finally we check for heterogeneity in the impact of treatment on individual policy preferences and attitudes using survey data. Since the answers are heavily skewed towards anti-refugee attitudes, we use logistic regression models in which the dependent variables take the value of 1 if the respondent chooses the response most critical towards refugees. We control for several individual-level attributes that have been shown to relate to anti-immigrant attitudes (Fitzgerald and Lawrence 2011), namely whether an individual has a high school degree, if they report that they are in a precarious economic situation, their self-reported gender, and if their settlement is within 25 km of a border (collected in the matrix $W_{ij}$). We include regional (NUTS 2) fixed-effects rather than county (NUTS 3) fixed-effects because we do not have survey participants from all 20 counties, and in several counties we only have untreated or treated observations. To test our hypothesis that treatment affects right-wing voters more than left-wing or non-partisan citizens, we introduce an interaction between treatment and whether the individual indicates a political preference for either Jobbik or Fidesz ($R_{j}$):

3 $$\begin{aligned} P(Y_{ij}=1)=\alpha +\delta _{1}T_{i}+\delta _{2}(T_{i}\times R_{j})+\beta _{3}R_{j}+\beta _{4}W_{ij}+\epsilon _{ij}. \end{aligned}$$

## Results

### Treatment and Referendum Voting Behavior

Table 1 presents our OLS models estimating results of the 2016 referendum on immigration at the settlement-level. As discussed in the previous section, our dependent variable is the number of ’no’ votes in the referendum as share of the total eligible voters. We first estimate the model controlling only for previous election results, population, and the socio-economic controls. In this first estimation, we find that treatment leads to a 3.5 percentage point higher share of no votes in a settlement. In a second step (Model 2) we introduce county-fixed effects and proximity to the border to control for the different geographic effects across the country. Here we observe a reduced though still significant effect of 1.7 percentage points. These findings support our first hypothesis, namely that short term exposure to refugees during the crisis leads to anti-refugee voting. Our estimates are similar to the 2 percentage point effect found by Dinas et al. (2019) in their study of far-right voting on Greek islands following the crisis. This is remarkable, considering the much more transient nature of the treatment in the Hungarian case.

**Table 1:** OLS regressions estimating the relationship between treatment and anti-refugee voting behavior in the 2016 Hungarian Quota Referendum

	*Dependent variable:*			
	Referendum no votes over eligible voting population			
	(1)	(2)	(3)	(4)
Treatment	0.035$$^{***}$$	0.017$$^{**}$$	0.022$$^{**}$$	
	(0.008)	(0.005)	(0.008)	
Mins (10) to treat.			$$-0.002^{***}$$	
			(0.0005)	
1–15 min to treat.				$$-0.027^{**}$$
				(0.011)
15–30 min to treat.				$$-0.031^{***}$$
				(0.009)
> 30 min to treat.				$$-0.036^{***}$$
				(0.009)
Fidesz share 2014	0.430$$^{***}$$	0.380$$^{***}$$	0.423$$^{***}$$	0.429$$^{***}$$
	(0.015)	(0.015)	(0.015)	(0.015)
Jobbik share 2014	0.212$$^{***}$$	0.203$$^{***}$$	0.217$$^{***}$$	0.212$$^{***}$$
	(0.021)	(0.021)	(0.021)	(0.021)
Population(log)	−0.022$$^{***}$$	−0.022$$^{***}$$	−0.022$$^{***}$$	−0.022$$^{***}$$
	(0.001)	(0.001)	(0.001)	(0.001)
Border < 25 km		−0.001		
		(0.003)		
Constant	0.341$$^{***}$$	0.330$$^{***}$$	0.359$$^{***}$$	0.377$$^{***}$$
	(0.016)	(0.016)	(0.017)	(0.019)
County FE	No	Yes	No	No
Controls	Yes	Yes	Yes	Yes
Observations	3142	3142	3140	3142
Adjusted R$^{2}$	0.367	0.458	0.372	0.367
Resid. Std. Error	0.073	0.067	0.073	0.073
F Statistic	260.6$$^{***}$$	103.2$$^{***}$$	233.1$$^{***}$$	203.2$$^{***}$$

In Model 4 treatment serves as a reference category

*p < 0.10, **p < 0.05, ***p < 0.01

Models 3 and 4 in Table 1 test the effect of distance from treatment. In Model 3 we observe that anti-refugee voting decreases roughly .2% for every ten additional minutes travel time from the nearest treatment. Model 4, in which treated towns serve as the reference category, suggests that treatment spills over to neighboring towns. These models support the notion that the effect of short term exposure on anti-refugee voting behavior is tightly concentrated in and around the treated settlements.

### Change in Party Vote Shares

Table 2 presents the results of our difference-in-differences estimations of the change in Fidesz, Jobbik, and combined Fidesz and Jobbik (right-wing, for short) vote shares between the 2014 and 2018 parliamentary elections. The results suggest that there was no significant overall effect of treatment on votes of the right-wing as a whole. However, we see a redistribution of votes within the camp: while Jobbik gained roughly two percentage points in treated settlement, Fidesz lost two percentage points, compared to settlements through which refugees did not travel. We also report a placebo test of the same models using data from the 2010 and 2014 parliamentary elections to test the parallel trends assumption. We do not observe the same redistribution of votes from Fidesz to Jobbik across the previous elections. We find similar effects when extending the treatment to settlements within 5 km of our original treatment and exclude settlements 5–20 km away from the matching (Selb and Munzert 2018), reported in the appendix (Table  9).

**Table 2:** Difference-in-differences estimation results and placebo tests. We analyzed the change in vote shares between 2014 and 2018 for Jobbik, Fidesz, and Jobbik and Fidesz (right-wing) together

Change in vote shares of	Fidesz	Jobbik	right-wing (F+J)
2014–2018			
After	0.070***	− 0.033***	0.042***
	(0.002)	(0.01)	(0.002)
Treatment	− 0.040***	− 0.032***	− 0.072***
	(0.015)	(0.10)	(0.002)
After $\times$ Treatment	− 0.021***	0.024***	** 0.003**
	**(0.005)**	**(0.003)**	**(0.004)**
2010–2014 (Placebo test)			
After	− 0.090***	0.060***	− 0.029***
	(0.002)	(0.001)	(0.002)
Treatment	− 0.031***	− 0.033***	− 0.065***
	(0.015)	(0.010)	(0.012)
After $\times$ Treatment	− 0.009*	0.001	− 0.008*
	(0.005)	(0.003)	(0.004)
R$^2$ (2014–2018)	0.16	0.06	0.21
R$^2$ (2010–2014)	0.23	0.22	0.18
$N$	3088	3088	3088

We find a significant redistribution of electoral support between Fidesz and Jobbik in treated settlements. We also report a placebo test supporting the parallel trends assumption. The regressions are run on a kernel-based propensity-score matched sample

*$$p<0.10$$, **$$p<0.05$$, ***$$p<0.01$$

These findings support our second hypothesis that Jobbik, as opposition party, would gain votes in treated settlements from the ruling Fidesz. They question the interpretation of previous results on short-term exposure and voting for the right as a consequence of the right-wing’s issuer-ownership of immigration rather than holding the government accountable. As a whole, the right-wing did not win more votes in exposed towns. In our context, the redistribution of votes within Hungary suggests an anti-government vote as Jobbik and Fidesz were competing with each other to take the more hardline anti-refugee position. Moreover, we note that the same diff-in-diff specification applied to the aggregated left-wing opposition results in 2014 and 2018 yields a null result (effect size: .002, p = .57).

More broadly, the different trajectory of Fidesz and Jobbik in treated settlements between 2014 and 2018 contrasts with the national results. Nationally Fidesz gained over four percentage points, while Jobbik lost more than one. In other words, right-wing voters in settlements exposed to the crisis punished the ruling party at the polls by voting for an alternative anti-refugee party, while elsewhere Fidesz expanded its support. We keep this question in mind as we contrast individual attitudes among left and right voters in treated settlements.

### Survey

Using data from a survey of the general population of Hungary in January 2016, between the peak of the crisis and the referendum, we consider how specifically exposure to refugees in the crisis may have changed the political opinions and policy preferences of Hungarians. We interact treatment with respondent’s party choice to see how this effect differs between left and right citizens. While we have no information on previous vote choices, citizens are asked about their current vote preference before the topic of immigration is broached in the survey.

One serious limitation of our survey analysis is that partisanship is self-reported and recorded after the crisis: it may be that exposure to refugees during the crisis moved individuals to the right, in particular those individuals who were especially influenced by their experiences. We test whether individuals in treated settlements were more likely to report support for a right-wing party and found no significant relationship. We report these results in the appendix (see Table 8). We also note that previous work on Hungary suggests that partisanship is increasingly consistent and polarized over time (Angelusz and Tardos 2011), rendering defection across the left and right camp less likely.

**Table 3:** Logistic regressions estimating the effect of treatment and association with the right on different anti-refugee attitudes

	Dependent variables: respondent anti-refugee response					
	No Refugees	L: Border	L: Culture	L: Money	W: Undoc	W: Culture
	(1)	(2)	(3)	(4)	(5)	(6)
Treatment	−0.25	0.004	−0.24	0.04	0.96$$^{**}$$	0.19
	(0.32)	(0.33)	(0.33)	(0.33)	(0.38)	(0.31)
Right-wing	0.31$$^{*}$$	0.43$$^{**}$$	0.06	0.07	0.23	0.37$$^{**}$$
	(0.17)	(0.19)	(0.17)	(0.17)	(0.19)	(0.17)
Treatment $$\times$$ R-W	0.27	1.52$$^{**}$$	0.13	1.07$$^{**}$$	1.39$$^{*}$$	1.43$$^{**}$$
	(0.46)	(0.70)	(0.47)	(0.52)	(0.83)	(0.58)
Border < 25 km	0.25	0.85$$^{**}$$	0.14	−0.27	0.88$$^{**}$$	1.05$$^{***}$$
	(0.28)	(0.35)	(0.28)	(0.28)	(0.37)	(0.32)
Highschool graduate	− 0.59$$^{***}$$	−0.35$$^{*}$$	−0.36$$^{**}$$	−0.38$$^{**}$$	−0.41$$^{**}$$	−0.37$$^{**}$$
	(0.17)	(0.19)	(0.17)	(0.18)	(0.19)	(0.18)
Precarious econ. situation	0.23	0.37$^{*}$	0.15	0.24	0.28	−0.02
	(0.18)	(0.21)	(0.18)	(0.18)	(0.21)	(0.18)
Male	0.30$$^{**}$$	−0.16	0.11	0.02	−0.05	0.01
	(0.15)	(0.17)	(0.16)	(0.16)	(0.17)	(0.16)
Constant	− 0.07	0.70$$^{***}$$	0.42$$^{*}$$	−0.39	0.65$$^{**}$$	0.23
	(0.23)	(0.26)	(0.24)	(0.24)	(0.25)	(0.24)
Regional FE	Yes	Yes	Yes	Yes	Yes	Yes
Observations	727	752	726	737	764	762
Log Likelihood	−485.41	−406.91	−479.17	−471.56	−408.24	−474.53
Akaike Inf. Crit.	998.81	841.82	986.34	971.11	844.48	977.05

L indicates the dependent variable is asking about a legal or policy preference, while W indicates the question concerns general worries about impact of the refugee crisis

$$^{*}$$p<0.1; $$^{**}$$p<0.05; $$^{***}$$p<0.01

Table 3 shows the impact of treatment on a battery of six different attitudinal questions. Besides the first question which asks respondents whether Hungary should accept refugees, all questions concern specific policy attitudes or respondents’ worries where responses may be more volatile. We present those variables in two groups: the first relates to questions about laws or policies that should be enacted in response to the crisis (Models 2, 3, and 4), and the second relates to how and why the respondent worries about the potential impact of the refugees (Models 5 and 6). Our translations of the questions are available in the Appendix (Table  5).

Model 1 measures which respondents are more likely to reject accepting any refugees at all, regardless of their origin. While we do not see a significant interaction effect, respondents who vote for Fidesz or Jobbik are more likely to reject all refugees. Model 2 to 4 analyze respondents’ support for different policies, namely the strengthening of border protection (2), a law obliging refugees to accept Hungarian culture (3) and additional money for integration (4). For consistency, we coded the dependent variable in Model 4 as rejection rather than support of additional money for the integration of refugees. We observe a significant and positive interaction effect for border security and the refusal to allocate more money to refugee integration. Model 5 and 6 analyze to which extent respondents are worried about the arrival of undocumented immigrants (5) and immigrants who belong to a different culture (6). Uniquely, Model 5 shows that respondents who live in treated settlements are more worried about the high number of undocumented immigrants coming to Hungary regardless of party, though the effect is stronger among right-wing voters. In contrast only right-wing voters express worry that arriving refugees come from different cultures. Arguably, left-wing voters and non-partisans also worry about changes in their settlement but draw different conclusions from this.

**Fig. 4:**
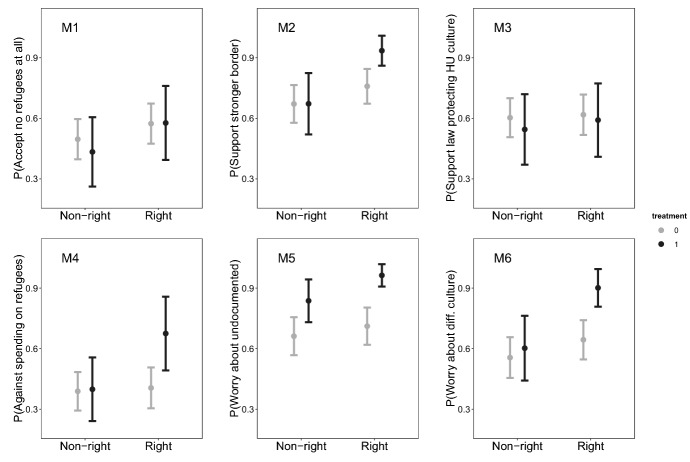
Conditional effects of interactions between partisanship and anti-refugee attitudes

To facilitate interpretation, we plot the conditional effects of our interaction terms in Fig. 4. Notably, in many of the models, the difference between treated and non-treated right-wing respondents is larger than the relatively small differences between left- and right-wing respondents in untreated settlements. We observe almost no change in model 1 and 3 which measure whether individuals reject accepting any refugees and whether they support a law that protects Hungarian culture.

Although not all interaction effects are statistically significant, we believe these results provide evidence that it was mostly right-wing citizens who hardened their position on immigration when exposed to refugees for a short period. While the limited size and scope of the survey does not allow us to dive deeper into the underlying mechanism, results from previous literature suggest these changes may be a consequence of a higher salience of the issue for right-wing voters (Neundorf and Adams 2018; Dennison 2019). Based on their lower attention to the issue, left-wing voters may attach less policy weight to their encounters with refugees. Overall, our survey-based analysis complements our difference-in-differences analysis, suggesting that right-wing parties mostly competed with each other to present tougher immigration policies.

## Conclusion

In this paper we related exposure to refugees during the 2015 crisis to political outcomes in Hungary. We find that exposure predicts anti-refugee voting in a national referendum on refugee quotas in 2016. Exposed settlements voted more for the far-right Jobbik party in the 2018 parliamentary elections, while the ruling Fidesz party, also right-wing and anti-refugee, lost votes. Overall, we see no aggregate gains by right-wing parties in treated towns. Finally, survey evidence suggests that exposure seems to galvanize anti-refugee attitudes only for right-wing partisans. In line with recent research on Western Europe (Dennison and Geddes 2019), we observe a mobilization of a pre-existing opposition to immigration instead of a change in underlying attitudes. Local experiences with refugees - whether it is direct encounters or merely stories about refugees passing by - seem to have increased the salience of the issue and thereby hardened the position of right-wing voters.

In contrast with previous work relating exposure to refugees to electoral outcomes, our first dependent variable more directly captures voting behavior on immigration issues. Hungary itself is also an interesting case as simultaneously one of the most xenophobic and least diverse countries in Europe. As Hungary has two significant right-wing anti-refugee parties, we can compare the effects of exposure on the support for the anti-refugee right in government and in opposition in the same context which marks a second departure from previous work. Thereby, our results contribute to the ongoing debate whether defection from governments during the crisis is driven by policy or anti-incumbent voting: while Fidesz and Jobbik presented similar anti-refugee stances, voters in exposed settlements defected from the governing party when presented with a credible anti-refugee alternative. Hence, without lowering the importance of issue ownership theory that likely motivates the choice of Jobbik as an alternative, our findings provide support for anti-incumbent voting in cases where voters do not need to compromise their policy preferences to punish incumbents. This suggests future research in other contexts where no radical right party was present may be promising.

Overall, our findings contribute to the ongoing discussion of how short-term exposure shapes the effect of the European refugee crisis. A growing body of research suggests that the length and conditions of exposure are decisive mediators in the formation of public opinion about refugees. We also note an interesting heterogeneity at the individual level based on partisanship. While Homola and Tavits (2017) suggest that left-wing voters become more tolerant with long-run exposure, we find that right-wing voters are significantly less tolerant after short term encounters.

Our results regarding the redistribution of votes within the right also lead us to caution against over-estimating the impact of exposure to refugees: While our effects are non-negligible and statistically significant, they primarily concern a shift within the right, not an expansion of the right-wing electorate. Additionally, these results ran counter to the development at the national level where Fidesz expanded its vote share based on an anti-refugee campaign.

These findings suggest some important policy implications. While most work on improving refugee integration outcomes focuses on long-term outcomes (Bansak et al. 2018) and studies targeted interventions (Lazarev and Sharma 2017), the finding that transient short term exposure inflames anti-immigrant attitudes indicates the value of investing in crisis management (Esses et al. 2017). Furthermore, the difference between the electoral results of Fidesz and Jobbik suggests that anti-refugee mobilization by governing parties may rather strengthen far-right opposition parties in exposed settlements. Some share of right-wing voters directly exposed to the crisis could not forgive even the quite credibly anti-refugee ruling government in Hungary. There are, it turns out, perhaps limits to exploiting a crisis, especially from a position of power (Table 10).

## Figures and Tables

**Fig. 5:**
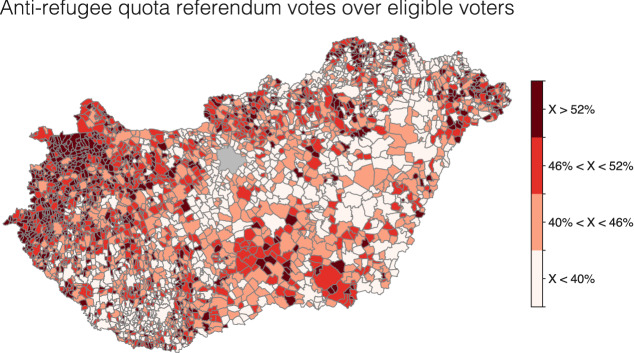
2015 Referendum anti-refugee resettlement quota voting quartile outcomes by settlement. Budapest (in gray) is excluded

**Fig. 6:**
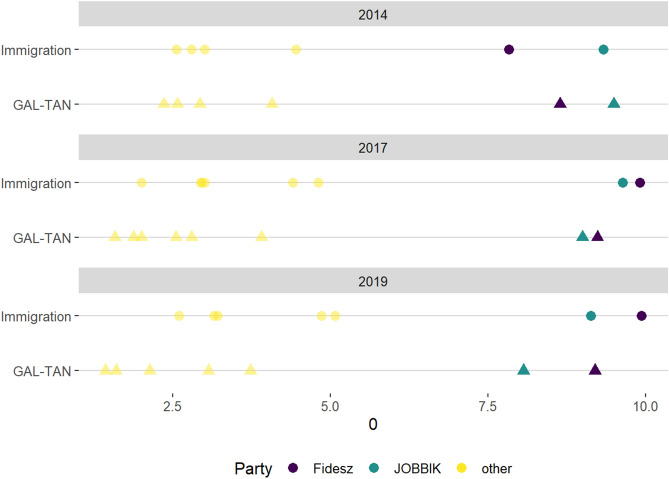
Policy positions of Hungarian parties on immigration and cultural issues more generally based on the Chapel Hill Expert Survey (Polk et al. 2017)

## Appendix

See FIgs. 5 and 6 and also Tables 4, 5, 6, 7, 8, 9, and 10.

**Table 4:** Summary statistics of Hungarian settlements

	N	Mean	St. Dev.	Min	Pctl(25)	Pctl(75)	Max
Ref. No/Eligible	3142	0.47	0.09	0.16	0.41	0.52	1.00
Fidesz share 2014	3142	0.51	0.11	0.10	0.44	0.58	1.00
Jobbik share 2014	3142	0.24	0.08	0.00	0.19	0.29	0.61
Treatment	3142	0.02	0.13	0	0	0	1
Border > 25 km	3142	0.23	0.42	0	0	0	1
Pct. Higher Edu.	3142	7.53	5.98	0.00	3.90	9.40	58.30
PC Income (1000s HUF)	3142	845	246	128	669	1,006	2,226
Pct. Unemployed	3142	0.06	0.03	0.00	0.04	0.08	0.24
Population (log)	3142	6.56	1.32	2.30	5.65	7.35	12.01
Mins. to Treatment	3140	5.67	2.94	0.00	3.44	7.74	14.31

Ref. No/Eligible refers to the key dependent variable in our first models: the ratio of voters voting against the EU refugee resettlement quota to the number of eligible voters in the settlement. Unless otherwise stated, all controls are taken from 2015

**Table 5:** Translated TARKI survey questions, January 2016

Variable in text	Question
No Refugees	Do you believe Hungary should accept every refugee, no refugees at all or some yes and others not?
L: Border	Do you agree that the Hungarian border should be strengthened?
L: Culture	Do you agree with the introduction of a law requiring immigrants to adhere to fundamental Hungarian cultural norms?
L: Money	Do you support increasing funding to refugees and immigrants living in Hungary for the purposes of integration (to facilitate their “new beginning” with residential, educational, and language-learning programs and assistance with finding work)?
W: Undoc	Are you worried that in a short period of time, many refugees and immigrants have arrived to Hungary unchecked (without documents)?
W: Culture	Are you worried that refugees and immigrants from different cultures and faiths are arriving to Hungary?
Precarious econ. situation	How would you rate your current economic situation?

**Table 6:** Summary statistics of survey respondents, January 2016

	N	Mean	SD
Treatment	772	0.14	0.34
Border < 25 km	772	0.10	0.30
Highschool Grad.	772	0.30	0.46
Precarious Econ. Sit.	768	0.27	0.45
Male	772	0.47	0.50
Right-wing	772	0.41	0.49
Left-wing	772	0.15	0.35
Support Fidesz	772	0.30	0.46
Support Jobbik	772	0.11	0.32
Want to Accept No Refugees	731	0.52	0.50
Support Stronger Border	756	0.73	0.44
Support Law Protecting HU Culture	730	0.58	0.49
Against Money for Refugee Integration	741	0.59	0.49
Worry about Undocumented Refugees	768	0.73	0.45
Worry about Cultural Differences	766	0.62	0.49
Met refugee in prev. 12 months	769	0.22	0.41
Know refugee/immigrant personally	770	0.03	0.17

**Table 7:** Logistic regression models predicting whether survey respondent has encountered refugee in previous 12 months (January 2016 Survey)

Dependent variable: respondent encountered refugee in previous 12 months				
	(1)	(2)	(3)	(4)
Treatment		1.937$$^{***}$$	1.928$$^{***}$$	1.860$$^{***}$$
		(0.224)	(0.230)	(0.236)
Respondent knows foreigner			2.684$$^{***}$$	2.699$$^{***}$$
			(0.531)	(0.533)
Border < 25 km				0.365
				(0.293)
Constant	−1.282$$^{***}$$	−1.650$$^{***}$$	−1.765$$^{***}$$	−1.796$$^{***}$$
	(0.087)	(0.106)	(0.111)	(0.114)
Log Likelihood	−402.415	−364.903	−347.316	−346.563
Akaike Inf. Crit.	806.830	733.806	700.633	701.126
$N$	769	769	768	768

Individuals living settlements exposed to the 2015 refugee crisis are significantly more likely to report encountering a refugee, even when controlling for knowing a foreigner personally or living close to a border

*$$p<0.10$$, **$$p<0.05$$, ***$$p<0.01$$

**Table 8:** Logistic regressions checking if survey respondents from treated settlements are more likely to report right-wing voting intentions, with the same individual-level controls as our primary regressions. We control for regional fixed-effects

	*Dependent variable:*	
	Respondent Right-wing Voter	
	(1)	(2)
Treatment		0.068
		(0.236)
Border < 25 km	−0.270	−0.285
	(0.265)	(0.270)
Highschool graduate	−0.243	−0.247
	(0.170)	(0.171)
Precarious econ. situation	−0.691$$^{***}$$	−0.691$$^{***}$$
	(0.178)	(0.178)
Male	0.068	0.071
	(0.151)	(0.151)
Constant	0.033	0.027
	(0.213)	(0.215)
Regional FE	Yes	Yes
Observations	768	768
Log Likelihood	−508.383	−508.342
Akaike Inf. Crit.	1,038.766	1,040.683

$$^{*}$$p<0.1; $$^{**}$$p<0.05; $$^{***}$$p<0.01

**Table 9:** Difference-in-differences estimation results with treatment radius extended to 5 km and settlements between 5 and 30 km from treatment excluded from the matching

Change in vote shares of:	Fidesz	Jobbik	right-wing (F+J)
After	− 0.036***	− 0.022***	0.058***
	(0.004)	(0.01)	(0.002)
Treatment	− 0.052***	− 0.031***	− 0.056***
	(0.004)	(0.003)	(0.002)
After $\times$ Treatment	− **0.016*****	**0.019*****	** 0.002**
	**(0.006)**	**(0.004)**	**(0.643)**
R$^2$	0.14	0.04	0.15
$N$	2532	2532	2532

We analyzed the change in vote shares between 2014 and 2018 for Jobbik, Fidesz, and Jobbik and Fidesz (right-wing) together. We find a significant redistribution of support from Fidesz to Jobbik in treated settlements. The regressions are run on a kernel-based propensity-score matched sample

*$$p<0.10$$, **$$p<0.05$$, ***$$p<0.01$$

**Table 10:** Linear Probability Model estimating the effect of treatment and association with the right on different anti-refugee attitudes

	Dependent Variables: Respondent Anti-Refugee Response					
	No Refugees	L: Border	L: Culture	L: Money	W: Undoc	W: Culture
	(1)	(2)	(3)	(4)	(5)	(6)
Treatment	−0.06	0.003	−0.06	0.01	0.018$$^{**}$$	0.05
	(0.07)	(0.06)	(0.08)	(0.08)	(0.06)	(0.07)
Right-wing	0.07$$^{*}$$	0.08$$^{**}$$	0.01	0.02	0.04	0.08$$^{**}$$
	(0.04)	(0.03)	(0.04)	(0.04)	(0.03)	(0.04)
Treatment $$\times$$ R-W	0.06	0.17$$^{*}$$	0.03	0.21$$^{*}$$	0.10	0.22$$^{**}$$
	(0.11)	(0.09)	(0.11)	(0.11)	(0.09)	(0.10)
Border < 25 km	0.06	0.14$$^{**}$$	0.03	−0.06	0.13$$^{**}$$	0.20$$^{***}$$
	(0.07)	(0.06)	(0.07)	(0.06)	(0.06)	(0.06)
Highschool graduate	−0.14$$^{***}$$	−0.07$$^{*}$$	−0.09$$^{**}$$	−0.09$$^{**}$$	−0.08$$^{**}$$	−0.08$$^{**}$$
	(0.04)	(0.04)	(0.04)	(0.04)	(0.04)	(0.04)
Precarious econ. situation	0.06	0.06	0.04	0.05	0.04	−0.01
	(0.04)	(0.04)	(0.04)	(0.04)	(0.04)	(0.04)
Male	0.07$^{*}$	−0.03	0.02	0.004	−0.01	0.005
	(0.04)	(0.03)	(0.04)	(0.04)	(0.03)	(0.03)
Constant	0.41$$^{***}$$	0.79$$^{***}$$	0.57$$^{***}$$	0.64$$^{***}$$	0.72$$^{***}$$	0.59$$^{***}$$
	(0.06)	(0.05)	(0.06)	(0.06)	(0.05)	(0.05)
Observations	727	752	726	737	764	762
R$^{2}$	0.05	0.08	0.04	0.07	0.09	0.08
Adjusted R$^{2}$	0.03	0.06	0.03	0.05	0.07	0.06
Residual Std. Error	0.49	0.43	0.49	0.48	0.43	0.47
F Statistic	2.80$$^{***}$$	4.88$$^{***}$$	2.45$$^{***}$$	4.22$$^{***}$$	5.65$$^{***}$$	4.70$$^{***}$$

L indicates the dependent variable is asking about a legal or policy preference, while W indicates the question concerns general worries about impact of the refugee crisis

$$^{*}$$p<0.1; $$^{**}$$p<0.05; $$^{***}$$p<0.01

## Footnotes

1 Additionally, in a study of extended contact Homola and Tavits (2017) find that contact with immigrants only reduces threat perceptions significantly for individuals with left-wing attitudes because of their higher openness to change. When exposure is passing, we suggest that the opposite mechanism may apply. A brief experience may not impact left partisans but reinforce the perception that outsiders are threatening which is associated with resistance towards change (Homola and Tavits 2017).

2 For example: “Do you think that Hungary could be the target of an act of terror in the next few years?” and “We hear different views on the issue of immigration. There are some who think that economic migrants jeopardise the jobs and livelihoods of Hungarians. Do you agree?”

3 Previous research has noted that, different from Western Europe, criticism of the EU from the (radical) right in Eastern Europe hardly targets the EU as an institution but EU interventions for minority rights (Bustikova 2017, 573).

4 In terms of their policy positions on immigration, the Chapel Hill Expert Survey (Polk et al. 2017) locates Jobbik to the right of Fidesz in 2014, both as almost indistinguishable with Fidesz slightly to the right in 2017 and Fidesz decidedly more right-wing than Jobbik in 2019, see Fig. 6 in the Appendix.

5 The results presented are robust to considering geographic distance instead.
